# Muscles are barely required for the patterning and cell dynamics in axolotl limb regeneration

**DOI:** 10.3389/fgene.2022.1036641

**Published:** 2022-10-10

**Authors:** Yan Hu, Xiangyu Pan, Yu Shi, Yuanhui Qiu, Liqun Wang, Prayag Murawala, Yanmei Liu, Wanjin Xing, Elly M. Tanaka, Ji-Feng Fei

**Affiliations:** ^1^ Inner Mongolia Key Laboratory for Molecular Regulation of the Cell, College of Life Sciences, Inner Mongolia University, Hohhot, China; ^2^ Department of Medical Research, Guangdong Provincial People’s Hospital, Guangdong Academy of Medical Sciences, Guangzhou, China; ^3^ Guangdong Cardiovascular Institute, Guangdong Provincial People’s Hospital, Guangdong Academy of Medical Sciences, Guangzhou, China; ^4^ Key Laboratory of Brain, Cognition and Education Sciences, Ministry of Education, Institute for Brain Research and Rehabilitation, South China Normal University, Guangzhou, China; ^5^ MDI Biological Laboratory, Bar Harbor, ME, United States; ^6^ Clinic for Kidney and Hypertension Diseases, Hannover Medical School, Hannover, Germany; ^7^ Research Institute of Molecular Pathology (IMP), Vienna BioCenter (VBC), Vienna, Austria; ^8^ Department of Pathology, Guangdong Provincial People’s Hospital, Guangdong Academy of Medical Sciences, Guangzhou, China

**Keywords:** axolotl, limb regeneration, salamander, single cell RNA sequencing, knockout, *Pax7*

## Abstract

Regeneration of a complex appendage structure such as limb requires upstream and downstream coordination of multiple types of cells. Given type of cell may sit at higher upstream position to control the activities of other cells. Muscles are one of the major cell masses in limbs. However, the subtle functional relationship between muscle and other cells in vertebrate complex tissue regeneration are still not well established. Here, we use *Pax7* mutant axolotls, in which the limb muscle is developmentally lost, to investigate limb regeneration in the absence of skeletal muscle. We find that the pattern of regenerated limbs is relative normal in *Pax7* mutants compared to the controls, but the joint is malformed in the *Pax7* mutants. Lack of muscles do not affect the early regeneration responses, specifically the recruitment of macrophages to the wound, as well as the proliferation of fibroblasts, another major population in limbs. Furthermore, using single cell RNA-sequencing, we show that, other than muscle lineage that is mostly missing in *Pax7* mutants, the composition and the status of other cell types in completely regenerated limbs of *Pax7* mutants are similar to that in the controls. Our study reveals skeletal muscle is barely required for the guidance of other cells, as well the patterning in complex tissue regeneration in axolotls, and provides refined views of the roles of muscle cell in vertebrate appendage regeneration.

## Introduction

The limb is a complex organ contains multiple types of tissues and cells, such as skin, bone, cartilage, muscle, nerve, fibroblast and tendon. Reconstructing such a structure, that is generally incapable in mammals, is a technical challenging issue. One of the major obstacles is the lack of sufficient and systematic understanding of the interaction and the upstream and downstream relationship of the cell types involved in complex tissue regeneration. Axolotl is a tetrapod vertebrate that is able to regenerate diverse organs including the limb. Importantly, the cell/tissue composition and the structure of axolotl limb are similar to that in higher vertebrates, and the lost cell/tissue types and the structure could be precisely reproduced during regeneration, that very much resemble the original limb before amputation or injury (Sessions et al., 1989; Brockes and Kumar, 2005; Nacu and Tanaka, 2011; Flowers et al., 2017; Haas and Whited, 2017).

Using various amphibian appendage regeneration models, previous studies showed that several types of cells and tissues, such as nerves, macrophages and apical epithelial cap cells play essential roles in mediating the injury responses or stimulating proliferation of many other cell types during complex tissue regeneration (Todd, 1823; Thornton, 1960; Makanae and Satoh, 2012; Godwin et al., 2013; Stocum, 2017; Aztekin et al., 2019). Limbs fail to regenerate properly upon denervation. Without nerve tissues, the wound healing process seems normal, but blastema formation, proliferation of the progenitors that participate in regeneration is impaired (Todd, 1823; Makanae and Satoh, 2012; Stocum, 2017). Several studies identified that nerve stimulates progenitor activities through nerve-secreted factors including anterior gradient (AP), BMPs, FGFs, NRG1 and a-MSH (Kumar et al., 2007; Satoh et al., 2010; Makanae et al., 2014; Farkas et al., 2016; Zhang et al., 2018). Furthermore, recent studies revealed that macrophages are also critical in successful complex tissue regeneration. Chemical depletion of macrophages leads to the failure of limb regeneration, but not the wound closure (Godwin et al., 2013; Stocum, 2017). In addition, apical epithelial cap has been reported to be one of the early formed signaling center to guide the underneath blastema cells proliferation (Thornton, 1957; Thornton, 1960; Makanae and Satoh, 2012; Stocum, 2017). These works suggest that some tissues/cells, such as nerves, macrophages, apical epithelial cap cells playing upstream regulatory roles in regeneration events, either producing a regeneration-permissive environment or triggering directly progenitor proliferation, to guide the downstream regenerative responses of other cell types.

Muscle is one of the major cell types in appendages including limbs. Limb muscles are mainly developed from muscle precursors, which are originated from somatic mesoderm (Christ and Brand-Saberi, 2002). Transcription factors *Pax3* and *Pax7* had been reported playing essential role on the maintenance of muscle precursors (Franz et al., 1993; Bober et al., 1994; Relaix et al., 2004; Relaix et al., 2005; Wang and Rudnicki, 2011). In mammals, knockout of the *Pax3*, but not the *Pax7* gene led to the migration failure of muscle precursor into the limb buds, thus causing the loss of limb muscle (Franz et al., 1993; Bober et al., 1994; Relaix et al., 2004). Recently, the conservative role of *Pax3* on limb muscle development was also identified in salamander newt *Pleurodeles waltl*, in which mutation of *Pax3* results in the loss of limb muscle and lethality of early larvae (Elewa et al., 2017), consistent with the phenotypes observed in mammals (Bober et al., 1994). However, the *Pax3* gene is genetically lost in the axolotl genome, but surprisingly its function was taken over by the paralog *Pax7* gene. Knockout of the *Pax7* gene in axolotls gives rise to multiple defects that covers the scope of the *Pax3* mutant phenotypes, including loss of limb muscle phenotype (Nowoshilow et al., 2018).

To investigate the role of muscles in limb regeneration, about half a century ago, researchers managed to remove the limb skeletal muscles using surgery or X-ray irradiation approaches to produce skeletal muscle-reduced (−less) salamander limbs, then amputated and followed the limb regeneration process of those limbs (Carlson, 1972; Dunis and Namenwirth, 1977; Lheureux, 1983; Holder, 1989). It was showed that the overall morphology of the blastema and the final limb regenerates were not dramatically altered upon massive loss of muscle tissues (Carlson, 1972; Dunis and Namenwirth, 1977; Lheureux, 1983; Holder, 1989). Similar results were also observed during limb development (Kieny and Chevallier, 1979; Franz et al., 1993; Bober et al., 1994). Recently, taking the advantages of newt *Pax3* mutants, Elewa and colleagues investigated the limb regeneration using F0 *Pax3* mutant newts and showed that those animals can regenerate the limb after amputation, as previously reported (Elewa et al., 2017). Although all these earlier studies have suggested that muscle may not sit in the key upstream position in the complex tissue regeneration. However, a detailed characterization of the patterning and the unbiased dynamics of other cell types in muscle-free limb regeneration was still not well known.

Here, we take the advantage of a *Pax7* mutant axolotl line, in which the limb satellite cells and muscles are lost since early development (Nowoshilow et al., 2018), and test the role of muscles in limb regeneration at the cellular level. We find that in the absence of the limb muscle lineage, the patterning, early injury response and proliferation of major progenitors are relatively similar in the process of limb regeneration. Single-cell RNA sequencing (scRNA-seq) reveals that the cell composition in regenerated limb of the *Pax7* mutants is similar, except the near loss of cells in muscle lineage, to the controls. Our work provides direct evidences that muscle lineage does not carry patterning information and other instructions to regulate other cells/tissues activity, but solely responsible to its own in complex tissue regeneration.

## Results

### The morphology and pattern of regenerated limb is similar in the absence of skeletal muscle

**FIGURE 1:**
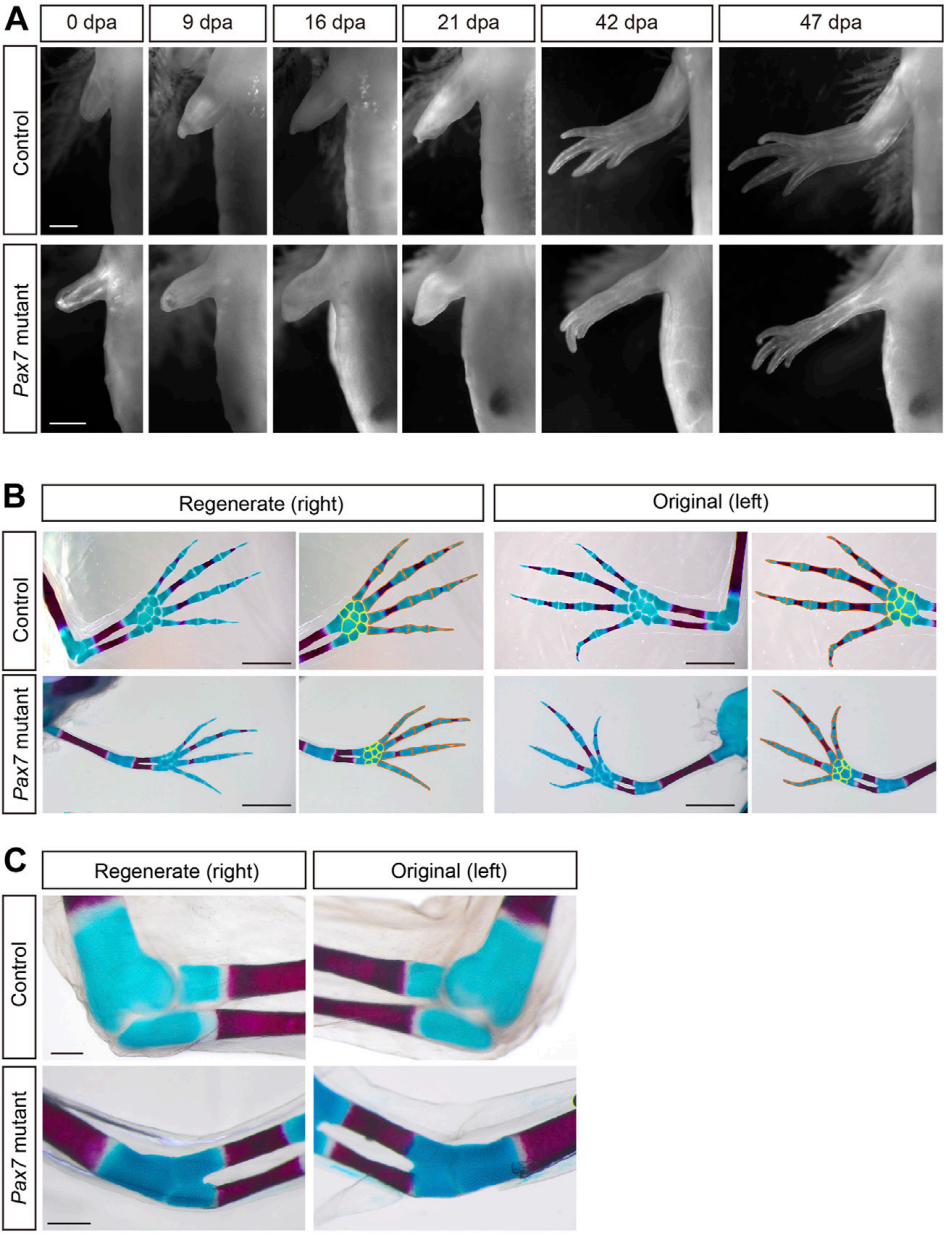
Morphology and patterning of limb regenerates in *Pax7* mutants. **(A)** Bright-field images of the limbs at indicated dpa, from 3-month-old controls and *Pax7* mutants. Scale bars, 1 mm. **(B)** Alcian blue and Alizarin red-stained fully regenerated (left panels) and un-operated (right panels) forelimbs from controls (upper panels) and *Pax7* mutants (lower panels). Amputations were carried out on 6-month-old animals, then allowed them to regenerate for 10-months for analysis. Hand part of each stained forelimb is presented at higher magnification, in which the red and yellow lines outline the phalange, metacarpal bones and carpal bones, respectively. Scale bars, 2 mm. **(C)** The elbow joint of regenerated (left panels) and un-operated (right panels) forelimbs from controls (upper panels) and *Pax7* mutants (lower panels). Scale bars, 500 μm.

To test the role of muscle tissues in complex tissue regeneration, we chose previously reported *Pax7* mutant axolotls, in which the limb skeletal muscles and satellite cells are completely lost due to the early developmental defects (Supplementary Figure S1) (Nowoshilow et al., 2018). When compared to controls, the overall size of limbs is slightly smaller in early juvenile *Pax7* mutants, due to the lack of limb muscle lineage. This discrepancy of the size of both limb and trunk increases with the proceeding of development, between the *Pax7* mutants and the controls (Nowoshilow et al., 2018). It is likely a side effect of the massive loss of trunk muscles at later stages (Nowoshilow et al., 2018), which disable *Pax7* mutants to acquire sufficient food and nutrition to support their growth. However, except for the muscle lineage, all other major tissues, such as bones, cartilages, nerves and connective tissues are to some extent, normally developed in early juvenile *Pax7* mutants (Supplementary Figure S2) (Nowoshilow et al., 2018). It allows us to investigate limb regeneration in the absence of muscle tissues. We first carried out limb amputation on both early juvenile healthy controls and muscle-free *Pax7* mutants, and followed the entire limb regeneration process. We found that the global morphology of blastema formation, growth and patterning of the limbs in *Pax7* mutants, based on the observation of over 30 individuals, are very similar to that in the controls (Figure 1A).

We further examined the patterning of regenerated limbs of the *Pax7* mutants and the controls in more detail. The bones and cartilages are generally used as an indication for the patterning of the limb regenerates (Maden, 1982; Thoms and Stocum, 1984). Alcian blue/alizarin red staining revealed that only the bone and cartilage structures distal to the amputation plane are regenerated, and the newly formed phalange, metacarpal and carpal bones are relatively proper patterned in *Pax7* mutants, which resembles the situation in the controls (Figure 1B). However, it appears that the size of both regenerated bone and cartilage structures were slightly reduced along proximal-distal and dorsal-ventral dimensions in *Pax7* mutants compared to the controls. It may due the body size of the *Pax7* mutants is dramatically smaller than that in the controls, and the size of the bone and cartilage structures proportionally reduced to match to the smaller body size in *Pax7* mutants. Amputation on both early (only cartilages in forelimbs) and late stage (calcified bone in forelimbs) juvenile axolotls yield similar regeneration phenotypes (Supplementary Figure S3, Figure 1B).

Based on the Alcian blue/alizarin red staining, we found that the elbow joint space between the upper and lower arms is disappeared in the *Pax7* mutant when compared to that in the control (Figure 1C). This defect is presented in both the original and regenerated limbs in *Pax7* mutant axolotls (Figure 1C). This finding is similar to developing mouse limbs devoid of muscle with a fusion of the elbow (or knee) joint (Kahn et al., 2009; Comellas et al., 2022). It suggests that the biomechanics generated by muscle is important in axolotls as it also is in mammals. Our results support that the muscle is not required for gross patterning of the limb, but is very important for the proper differentiation of the elbow joint, in both development and regeneration. Furthermore, we observed a slightly delayed regeneration (Figure 1A) and bone calcification (Figure 1B) in *Pax7* mutants compared to the controls. At 42 days post amputation, most of the *Pax7* mutants had three fingers, whereas controls had four fingers (Supplementary Table S1). The number of fingers in *Pax7* mutants also reached four in the fully regenerated limbs. So that we concluded a slightly delayed regeneration in *Pax7* mutants compared to the controls*.* It is in line with the delayed limb developmental phenotype in late stage *Pax7* mutants (Nowoshilow et al., 2018). Presumably, the muscle defects result in the disability of mutants to eat enough Artemia food, therefore lack of enough nutrition to support regeneration. These results suggested that the skeletal muscles do not harbor the positional information and likely other crucial cues to guide the patterning in a complex tissue regeneration.

### Macrophage recruitments in early injury response do not require muscles

**FIGURE 2:**
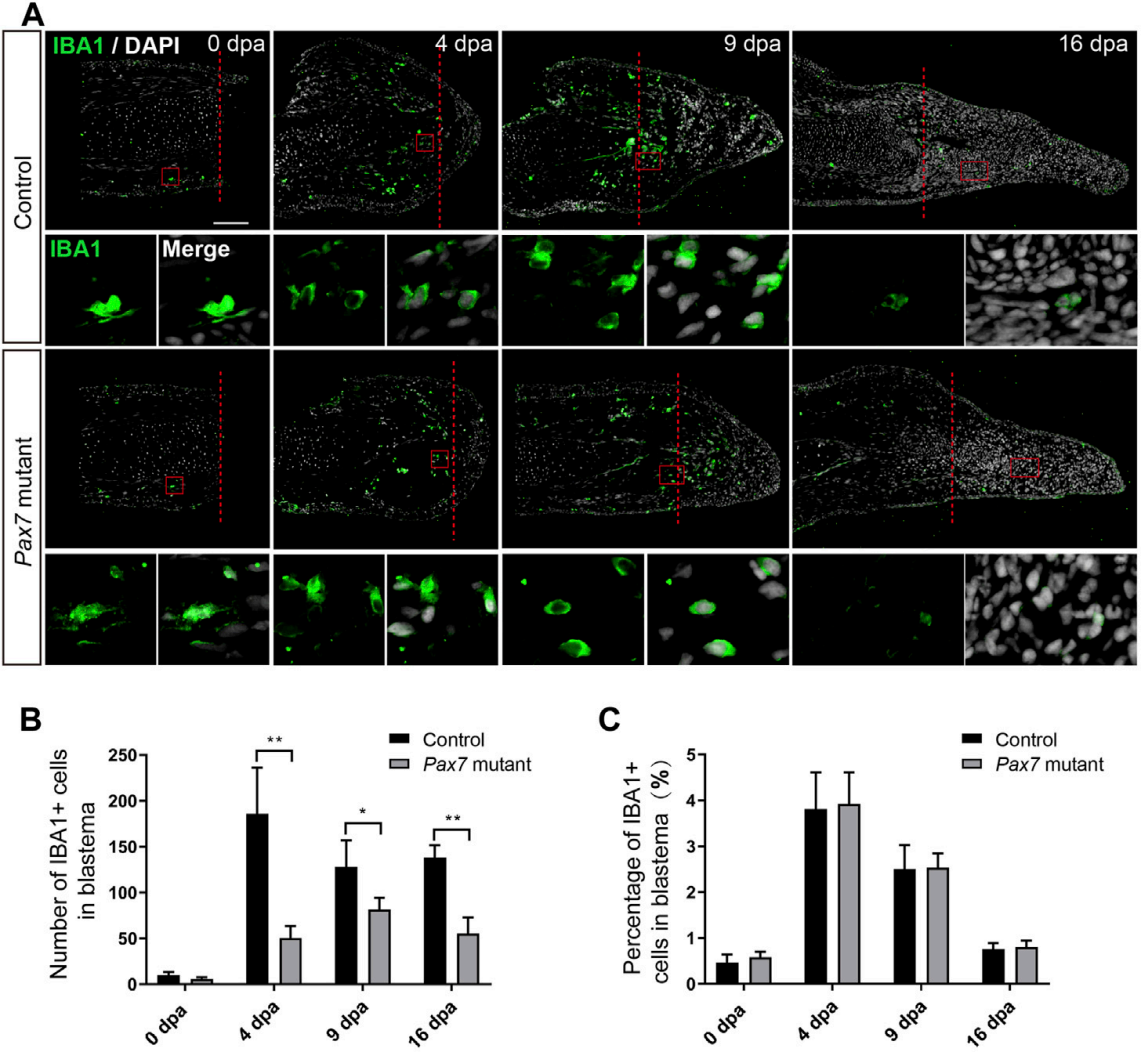
Macrophage dynamics in limb regeneration of *Pax7* mutants. **(A)** Immunofluorescence images of IBA1 (green) and DAPI (white) in limb longitudinal-sections, at 0, 4, 9 and 16 dpa, from control and *Pax7* mutant animals. Boxed regions are shown at higher magnification as separated channels. Red dashed lines, amputation planes. Scale bars, 200 μm. **(B)** and **(C)** Quantification of the absolute number **(B)** and the percentage **(C)** of IBA1 positive cells, within the areas of the newly formed blastema combined 500 μm zone proximal to the amputation plane, at 0, 4, 9 and 16 dpa, from controls (*n* = 4) and *Pax7* mutants (*n* = 4). Data are presented as mean ± sem, *p < 0.05, **p < 0.01.

To figure out whether there are any changes on early injury responses in the absence of muscles, we chose the immune response, one of the early injury-induced events that has been reported to play essential role in limb regeneration (Godwin et al., 2013), and examined the immune cell behavior in *Pax7* mutants. We carried out amputation on the upper limbs in both of the *Pax7* mutants and the controls, and collected samples at 0, 4, 9, and 16 day post amputation (dpa), which covers from early blastema to early differentiation stages. We used IBA1 as a marker to identify the macrophage cells on the limb longitudinal sections. The immunohistochemistry results showed that IBA1-positive macrophages are enriched to the amputation plane at early regeneration phase (4 dpa) in the controls, gradually declined at 9 dpa and finally returned back to about normal level at 16 dpa, as that observed at 0 dpa (Figures 2A–C). Although the absolute number of IBA1-positive cells is reduced throughout the entire regeneration stages in *Pax7* mutants compared to the controls (Figure 2B), when presented as a percentage over all blastema cells, the proportion and dynamics of macrophages in limb regeneration of *Pax7* mutants resemble the situation in the controls (Figure 2C). This observation suggested that the muscles likely are not involved in the recruitment of macrophage at early regeneration stages.

### Fibroblast proliferation is not affected in the absence of skeletal muscle in limb regeneration

**FIGURE 3:**
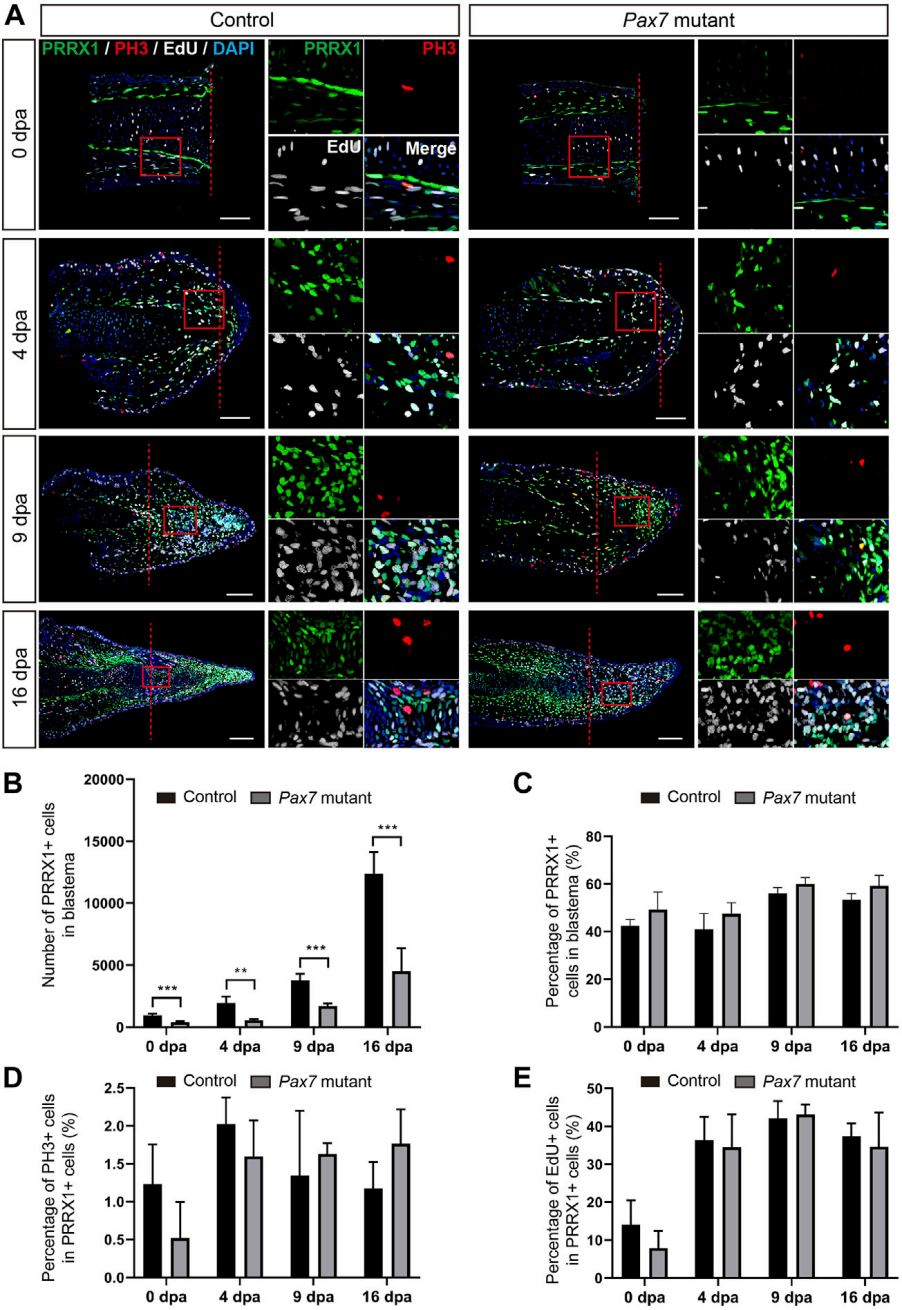
Fibroblast dynamics during limb regeneration in the absence of muscles. **(A)** Immunofluorescence images of PRRX1 (green), PH3 (red), combined with EdU (white) and DAPI (blue) staining in limb longitudinal-sections, at 0, 4, 9 and 16 dpa, from control and *Pax7* mutant animals. Boxed regions are shown at higher magnification as separated channels. Red dashed lines, amputation planes. Scale bars, 200 μm. **(B)** and **(C)** Quantification of the absolute number **(B)** and the percentage **(C)** of PRRX1 positive cells, in the areas of the newly formed blastema combined 500 μm zone proximal to the amputation plane, at 0, 4, 9 and 16 dpa, from controls (*n* = 4) and *Pax7* mutants (*n* = 4). Data are presented as mean ± sem, **p < 0.01, ***p < 0.001. **(D,E)** Quantification of the percentage of PH3- **(D)** and EdU- **(E)** positive cells, in PRRX1-positive cells quantified in **(B)**.

We next checked whether muscles play any role on the proliferation of blastema cells to guide the blastema formation. Previous studies have shown that limb blastema is the mixture of lineage-restricted progenitors (Kragl et al., 2009). Connective tissue (fibroblast) is one of the major cell populations comprising the blastema, and has been reported to play a critical role in axolotl appendage regeneration (Stocum, 1975; Satoh et al., 2008; McCusker and Gardiner, 2013; Wu et al., 2013; Phan et al., 2015; Currie et al., 2016; Gerber et al., 2018; Leigh et al., 2018; Lin et al., 2021). Therefore, we chose fibroblasts as a target for the examination of cell expansion and cell cycle dynamics in limb regeneration without muscles. We performed limb amputation and sample collection on the *Pax7* mutants and the controls as above described, additionally introducing a single pulse of EdU prior to sample collection. We then carried out immunohistochemistry using antibodies again PRRX1 (fibroblast) and Phospho-histone H3 (PH3, mitotic cells) on longitudinal sections, combined with EdU detection, to highlight fibroblast and reveal their dynamics (Figure 3A). Immunohistochemistry and quantification of PRRX1-positive cells showed that the absolute number of PRRX1-positive fibroblasts is less in the *Pax7* mutants throughout the process of regeneration, when compared to the controls (Figures 3A,B). It may represent the ground status of PRRX1-positive fibroblasts reduction in *Pax7* mutants prior to limb amputation, since the size of the limb is smaller in *Pax7* mutants. However, with the proceeding of regeneration, the number of fibroblasts gradually increase in blastema in both *Pax7* mutants and controls. And the proportions of fibroblasts in blastema are comparable (slightly increased, but statistically not significant) at each regeneration time point in *Pax7* mutants, when compared to the controls (Figure 3C), that is in line with the fact that myocytes and satellite cells comprise only a relatively small proportion of cells in early limb blastemas (Supplementary Figure S4). Furthermore, EdU-positive or PH3-positive cells, which label fibroblasts at S- or M-phases of the cell cycle respectively, also occupied similar ratio in the *Pax7* mutants and controls at all regeneration stages analyzed (Figures 3D,E). These results indicated cell cycle dynamics of PRRX1-positive fibroblasts are not dramatically affected in the absence of muscles during limb regeneration.

### Full spectrum of cell types, except muscle lineage are regenerated in *Pax7* mutant limbs

**FIGURE 4:**
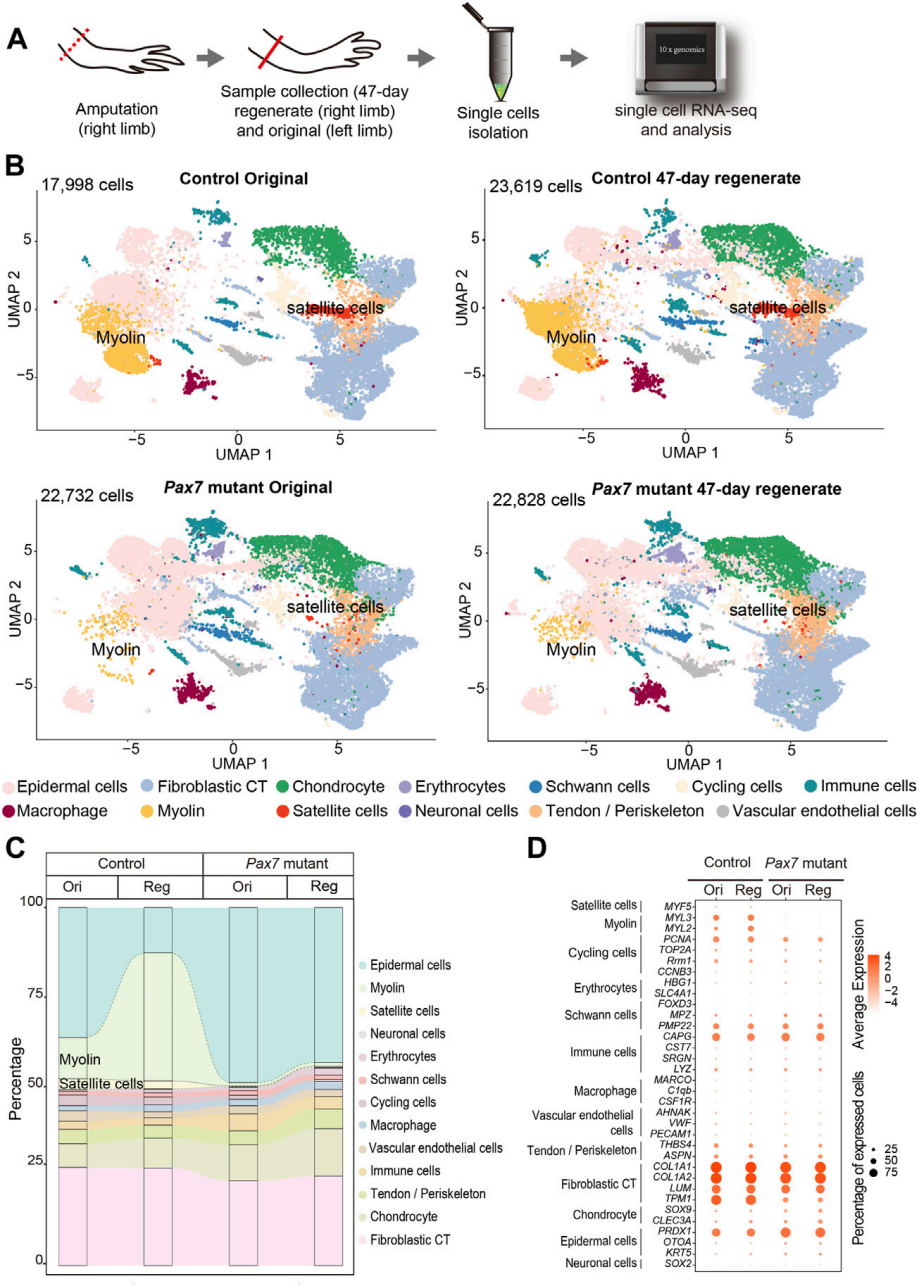
Integrated transcriptional cell state atlas of the axolotl limb from the 47-day regenerate and original in both controls and *Pax7* mutants. **(A)** Scheme of the scRNA-seq workflow used for the limb tissues collected from the 47-day regenerate (right limb) and original (left limb) in both controls and *Pax7* mutants. Dotted line, amputation plane; line, the position of sample collection. **(B)** UMAP visualization of the scRNA-seq data from four sampling stages. The cell-type annotation is determined by published cell-lineage specific markers. Two muscle-related cell types are highlighted. **(C)** Sankey plot showing the percentage of all 13 cell types from the 47-day regenerate and original limbs in both controls and *Pax7* mutants. **(D)** Dot plot visualizing the expression of marker genes of all 13 cell types shared between the controls and *Pax7* mutants. The circle size represents the percentage of cells at each sample expressing the gene, and color represents the average expression level.

To determine whether other relevant cell types, in addition to fibroblasts, respond properly during regeneration in the absence of muscles, we systematically examined cell type composition in fully regenerated forelimbs from both *Pax7* mutants and controls. To this end, we collected uninjured and 47-days regenerated forelimbs from *Pax7* mutants and controls, isolated single cells and carried out scRNA-seq, which allowing unbiased identification of cell types (Figure 4A). In total, we obtained 87,177 high-quality cells for single cell transcriptome and cell type identity analysis. After clustering all cells and reflecting them in uniform manifold approximation and projection (UMAP), we identified 41 putative cell populations. We then used typical cell type markers identified previously to assign each cluster (Gerber et al., 2018; Leigh et al., 2018; Li et al., 2021; Lin et al., 2021; Qin et al., 2021). The finally defined clusters belong to 13 cell types which cover all major limb cell types (Figure 4B), such as fibroblastic connective tissue (CT) cells, macrophages, Schwann cells, and chondrocytes. We found that in the uninjured limbs, except satellite and myocytes, all other cell types are identified in both *Pax7* mutants and controls (Figures 4B,C). Moreover, cell type composition in the 47-days regenerated limbs recapitulates the situation in uninjured limbs, from which only the cell types in muscle lineage abruptly decline in the *Pax7* mutant limb regenerates (Figures 4B,C). Meanwhile, we also observed that the proportions of the majority of cell types are nearly unchanged in the *Pax7* mutants compared to the controls, except for the increased epidermal cell level and nearly loss of skeletal myocyte in the *Pax7* mutant regenerates (Figures 4B,C). The dramatic increase of the ratios of epidermal cells in the *Pax7* mutants, is likely caused by the increased proportion of skin tissues from the loaded samples. As shown in our data, the size of the limbs from the *Pax7* mutants is smaller than that from the controls. Therefore, we collected more limb samples from the *Pax7* mutants for cell dissociation in this single cell sequencing experiment (Supplementary Table S2). In this case, the tissue volume used for single cell sequencing experiment is similar between the *Pax7* mutants and controls. Considering each *Pax7* mutant limb as a unit is smaller in size than that from the control, a higher proportion of skin tissues/cells were loaded in the *Pax7* mutants than the controls for single cell sequencing analysis.

We next performed Pearson correlation between each cell type from the original and regenerated limbs of the controls and the counterpart of the *Pax7* mutants, separately (Supplementary Figure S5). Each cell type between the control and the *Pax7* mutant animals has a high correlativity. Furthermore, we analyzed differentially genes of Fibroblast CT, Epidermal cells and Chondrocyte between the controls and the *Pax7* mutants of original and regenerated limbs, separately. Our results showed that there has no significant difference between the controls and *Pax7* mutants of original and regenerated limbs in these three major cell types. Furthermore, we chose several major marker genes of all the 13 cell types, and analyzed the expression of these genes in all major identified cell types in both *Pax7* mutants and controls. We found that cells in *Pax7* mutants largely share common gene expression patterns with the cells in controls (Figure 4D). Our data suggest that the deficiency of limb muscles do not change the cell identities and states at a systematic molecular level.

**FIGURE 5:**
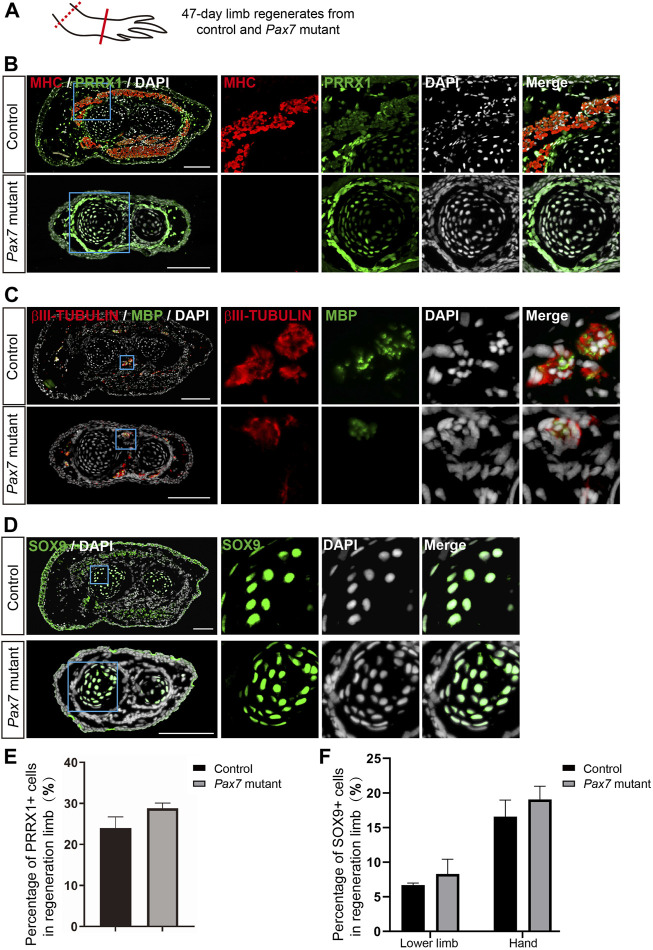
Examination of cell types in limb regenerates in *Pax7* mutants. **(A)** Scheme of the sample collection. Dotted line, amputation plane; line, the position of analyzed section. **(B–D)** Immunofluorescence images of MHC (red), PRRX1 (green) and DAPI (white) **(B)**; βIII-TUBULIN (red), MBP (green) and DAPI (white) **(C)**; and SOX9 (green) and DAPI **(D)**, in cross-sections, from 47-days fully regenerated control and *Pax7* mutant limbs. Boxed regions are shown at higher magnification as separated channels. Scale bars, 200 μm. **(E,F)** Quantification of the percentage of PRRX1 **(E)** and SOX9 **(F)** positive cells, in the limb from controls (*n* = 3) and *Pax7* mutants (*n* = 3). Data are presented as mean ± sem.

Moreover, we performed immunohistochemistry on cross sections of 47-days limb regenerates from both *Pax7* mutants and controls, using antibodies against MHC, PRRX1, MBP, βIII-tubulin and SOX9, which labels muscle cells, fibroblastic CT cells, Schwann cells, neuronal cells and chondrocytes, respectively. We found that, in consistent with the scRNA-seq data, all these identified cell types are all present in the regenerated limb in *Pax7* mutants. And the distribution of each cell type in limb regenerates resembles its location in original uninjured limb (Figures 5A–D, Supplementary Figure S6). Furthermore, we quantified the proportions of PRRX1 and SOX9 positive cells in fully regenerated limbs and found that the proportion of PRRX1 positive Fibroblasts in regenerated lower limb is slightly higher, but not statistically significant in *Pax7* mutants when compared to the controls (Figure 5E). The proportion of SOX9 positive cells is similar in the lower limb and hand of *Pax7* mutants compared to the controls (Figure 5F). It may be due to the loss of muscle tissues that indirectly causes the proportion change of these cell types in regenerated limb between two samples. Overall, these results reveal that all other cell types in limb regenerate relatively proper without the support of muscle tissues. Therefore, we conclude that muscles play minor regulatory roles on the regeneration of other cells types in complex tissue regeneration in axolotls.

## Discussion

In this study, taking the advantages of *Pax7*-CRISPR mutant axolotls in which harbor no limb muscles, we characterized the dynamics of other cells in limb regeneration. We found that muscle tissues do not play a dominant role on regulating patterning, the early injury responses and proliferation of other cell types during regeneration. These data suggested that muscles sit at downstream position, meaning following the instructions of other key cell types, in a complex tissue regeneration.

### The role of muscle in axolotl limb regeneration

The mature limb consists of multiple tissues, including the epidermis, dermis, muscle, nerve, blood vessels and skeletal elements. Each tissue must regenerate coordinately according to its original pattern to restore functionality. Previous studies have found that some cell types, such as nerve and fibroblast CT cells are key for axolotl limb regeneration (McCusker and Gardiner, 2013; Gerber et al., 2018; Lin et al., 2021; Wells et al., 2021). Although classical studies in salamanders have shown that lack or reduction of muscle tissues does not dramatically change the gross morphology of regenerated limb (Carlson, 1972; Dunis and Namenwirth, 1977; Lheureux, 1983; Holder, 1989), the detailed patterning and dynamics of other cells during limb regeneration, under a muscle-less condition, remain poorly elucidated.

Furthermore, previous studies are based on non-genetic approaches (Carlson, 1972; Dunis and Namenwirth, 1977; Lheureux, 1983; Holder, 1989), either the surgical manipulation or X-ray irradiation combined with transplantation, to create muscle-less limb, then followed limb regeneration after amputation. In general, it is technically difficult to remove muscle completely, and indeed, the regenerated limbs still contain substantial amount of muscle tissues (Carlson, 1972; Dunis and Namenwirth, 1977; Lheureux, 1983; Holder, 1989). Until recently, CRISPR mediated gene knockout tools were successfully established in salamanders and used to create genetic muscle free limb models (Elewa et al., 2017; Nowoshilow et al., 2018), which allows further investigation of the role of muscle during limb regeneration, in a cleaner background. Studies in newts *Pax3* mutants elegantly demonstrated that limb lack of muscle could regenerate a relative normal muscleless limb (Elewa et al., 2017). Because the homozygous *Pax3* mutation in newt causes early embryonic lethality, therefore global phenotype analysis was carried out only in F0 chimeric individuals and no further detailed characterization performed (Elewa et al., 2017). In axolotls, the *Pax7* mutant lose completely the limb muscle, but the mutant *per se* can survive for up to 1 year (Nowoshilow et al., 2018). Therefore, here we used *Pax7* axolotl mutants to investigate the detailed patterning and kinetics of other cell types in muscleless limb regeneration. Based on these facts, we elucidated that muscles do not control the patterning and the dynamics, such as proliferation and differentiation of other cell types in regeneration, consistent with previous observation of the global phenotype of muscleless limb regenerates.

However, we identified the joint malformation defect in the *Pax7* mutant axolotls. As previous observed in mice, mechanical force mediated by the muscle contraction is critical for the proper cell fate commitment of joint progenitors. Loss of muscle tissues results in the developmental joint fusion phenotype (Kahn et al., 2009). We find similar joint defects in developing axolotls in the absence of muscle tissues. Interestingly, similar defects reappear during limb regeneration. It is worthwhile to further investigate whether joint defects identified were caused by similar mechanisms as identified in mammals.

Our multi-dimension studies revealed that lack of muscles does not affect the limb regeneration in axolotls. Firstly, the experimental data showed that muscles do not retain patterning information. Secondly, muscle tissues do not play a dominant role on regulating the early injury responses or proliferation of other cell types during regeneration. Overall, our data indicate that muscles locate at downstream pyramid in complex tissue regeneration.

### The lineage of muscle regeneration

During axolotl limb regeneration, the muscle precursors are the major source of the skeletal muscle regeneration (Fei et al., 2017). But in salamander newts, previous studies showed that muscle de-differentiation derived progenitor cells contribute substantially to muscle regeneration (Sandoval-Guzmán et al., 2014). Whether muscle precursors contribute, and to which extent, to muscle regeneration are still not determined in newts. During development, however, there are multiple origins of muscles. Both muscle precursors and fibroblast contribute to skeletal muscles formation (Kieny et al., 1988). In our sc-RNA seq data, we found there are minor amount of muscle cells in the *Pax7* limb regenerates. There are several possible explanations. Firstly, the *Pax7* mutants were established in the background of the commonly used d/d axolotl strain, that is not an inbred line. Therefore, the genetic background of each individual varies. For the sc-RNA experiment, we pooled limb samples from about fifty *Pax7* mutant axolotls for analysis. We do observe very few *Pax7* mutant individuals contain little muscle tissues (Supplementary Figure S7), from which may bring some muscle tissues into the samples analyzed. Secondly, the appeared muscles may come from trans-differentiation. During embryonic muscle development, fibroblasts have the potential to give rise to muscles (Kieny et al., 1988). Several recent studies have revealed that fibroblasts are capable of trans-differentiating into skeletal muscles *in vitro* under given conditions (Boularaoui et al., 2018; Xu et al., 2020). Although still lack evidence, we cannot rule out the possibility that these very few muscles observed in *Pax7* limb regenerate are originated from fibroblasts or other cell types.

### The role of muscle during evolution

Furthermore, recent studies revealed that muscle plays critical roles in regulating regenerative responses in planaria, particularly providing the positional instructions to surrounding cells and tissues, to maintain the proper regeneration (Witchley et al., 2013; Scimone et al., 2017). In axolotl limb regeneration, connective tissues, but not the muscles follow the rule of distal transformation (giving rise to tissues only distal to the amputation plane), and differentially express the transcription factor MEIS (a positional identity regulator) along the proximo-distal axis (Stocum, 1975; Satoh et al., 2008; Nacu et al., 2013; Wu et al., 2013; Phan et al., 2015). Here, we demonstrate that, as previously reported (Elewa et al., 2017), the overall patterning of limb regenerates is not affected in the absence of limb skeletal muscles. Interestingly, a recent study showed that muscles in planaria function to some extent as connective tissues (Cote et al., 2019). It will be necessary to directly test the role of connective tissues (fibroblasts) in vertebrates on positional determination in future, such as using genetic approach to ablate the population of fibroblasts, then follow the regeneration of complex tissues in the absence of connective tissues.

Overall, we found that lack of muscles does not affect the activities of macrophages immune response, proliferation of fibroblast and other cell types in limb regeneration process. Our work provided a fine cellular role of muscles, and revealed that muscles sit at lower position in the upstream and downstream coordination of complex tissues regeneration. It gives new insights into exploring essential components of tissues regenerating scaffolds, ranging from the macroscopic to the molecular scale in a upstream and downstream manner. Moreover, our finding may contribute to the realization of regenerative therapies by building biomaterial constructs *in vitro*.

## Materials and methods

### Axolotl care and operation

Animal experiments were carried out according to the guidelines of the ethics committee of Guangdong Provincial People’s Hospital. In this study, we used d/d and *Pax7*-CRIPSR mutant, strain tm (*Pax7* ^*4751V6D20/4754V6D20*^)^LABF^ axolotls (*Ambystoma Mexicanum*) (Nowoshilow et al., 2021), aging between 2.5 and 16 months for analysis. Axolotl larvae were kept individually in plastic containers with a change of fresh tap water once a day and fed artemia daily. Axolotls were anaesthetized with 0.01% ethyl-p-aminobenzoate (Benzocaine; Sigma) solution prior to amputation, imaging, EdU injection and sample collection. Amputation was carried out on right forelimb of axolotl larvae (about 3-month-old, except for one bone regeneration experiment, 6-month-old animals were used) by cutting at the mid-upper arm followed by trimming of the bone to allow proper blastema formation. Bright-field axolotl limb images were obtained using an Olympus stereomicroscope. Samples of uninjured limbs or limb regenerates were collected at the stages indicated for analysis. If applicable, a single pulse of EdU (at a dose of 10 mg/kg body weight) was introduced into axolotls by intraperitoneal injection, and kept for 3 h prior to sample collection. All samples collected were fixed in 1 × MEMFA (0.1 M MOPS, pH 7.4, 2 mM EGTA, 1 mM MgSO_4_ and 3.7% formaldehyde) for further analysis, unless specified.

### Alcian blue and Alizarin red staining

Samples were fixed in 4% PFA (4% paraformaldehyde prepared in phosphate buffer, pH 7.2) for at least 24 h for Alcian blue and Alizarin red staining. Briefly, after several washes in PBS, the internal organs of the fixed axolotls were carefully removed, then sequentially dehydrated in 25%, 50% and 70% ethanol solutions. Firstly, the samples were stained for cartilage tissues with 0.01% Alcian blue 8GX solution (prepared in ethanol and glacial acetic acid, 3:2) for approximately 48 h, then gradually rehydrated with 70%, 50% and 25% ethanol series, washed with distilled water for 24–48 h, followed by an enzymatically digested in 1% trypsin (MP Biomedicals, 153571) for 1–2 h at 37°C. After digestion, samples were rinsed in 1% KOH, then stained for bone tissues with 0.01% Alizarin Red solution (prepared in 1% KOH) overnight, followed by a series of 1% KOH washes until the samples look transparent. Lastly, the samples were dehydrated in 25%, 50%, 70%, 90% and pure ethanol series, then incubated sequentially in glycerol/ethanol series (1:3, 1:1, 3:1), and finally stored in glycerol at room temperature. Images were acquired with an Olympus SZX10 microscope (Olympus, Tokyo, Japan).

### Immunohistochemistry and EdU staining

We collected 10 μm-thick cryosections in accordance with standard procedure for Immunohistochemistry and EdU staining. When combined with EdU detection, we carried out firstly EdU staining using Click-iT EdU kit (Invitrogen, C10340) in accordance with the manufacturer’s instructions, then followed by a standard immunohistochemistry protocol as previously described (Fei et al., 2017). In brief, after PBS wash and permeabilization with PBST, slides were blocked in 5% serum prepared in PBST, then incubated with primary antibodies overnight at 4°C, followed by sequential PBST washes and secondary antibody (with DAPI, Sigma-Aldrich, D9542) incubation, and finally mounted using Mowiol 4-88 (Sigma-Aldrich, 9002-89-5) mounting medium after several PBST washes. Fluorescence images were acquired with an Olympus IX83 microscope (Olympus, Tokyo, Japan), using ×20 objectives and analyzed using FIJI. The following antibodies were used in this study, MBP (Genetex, GTX761141), PRRX1 (Gerber et al., 2018), βIII-TUBULIN (R&D, MAB1195), SOX9 (Chemicon, Ab5535), PAX7 (DSHB, AB528428), MEF2C (Santa Cruz, sc-365862), phospho-Histone H3 (Abcam, 10543), MHC (DSHB, A4.1025). Alexa 488-donkey-anti-mouse IgG (Jackson, 711-547-003), Alexa 555-donkey-anti-rat IgG (Invitrogen, SA5-10027), Alexa 647-donkey-anti-mouse IgG (Jackson, 715-607-003), CY3-donkey-anti-mouse IgG (Jackson, 715-165-151).

### Single cell dissociation of scRNA-seq

For each scRNA-seq experiment, 30–40 original or regenerating forelimbs were collected as a pool for single cell dissociation. Each sample was minced and digested in 1,000 μl 1 × Liberase (Roche) diluted in 0.8 × PBS- (without Mg^2+^/Ca^2+^) supplemented with 0.5 U/μl Dnase I (Thermo Scientific) at room temperature for approximately 30 min. The reaction was stopped by adding 10% FBS in PBS. Then, the cell suspension was filtered through a 70 μm cell strainer, followed by a centrifugation at 500 g for 5 min. Cells were resuspended in 2 ml 0.8 × PBS and filtered through a 30 μm cell strainer, followed by a centrifugation at 500 *g* for 5 min. Finally, the cells collected were resuspended in 0.5 ml 0.8 × PBS to generate a single-cell suspension used for the library construction.

### Library preparation and sequencing

The scRNA-seq library was constructed using the Chromium single-cell 3 prime v2 reagent kit (10 × Genomics) in accordance with the manufacturer’s instructions (https://support.10xgenomics.com/single-cell-gene-expression/index/doc/user-guide-chromium-single-cell-3-reagent-kits-user-guide-v2-chemistry). All libraries were further prepared based on the requirements of the Nova-seq6000 sequencing platform manufactured by MGI^®^. The DNA concentration was determined by a Qubit (Invitrogen^®^). Then, samples with 2 pmol of nucleotides were pooled to generate single-strand DNA circles (ssDNA circles). DNA nanoballs (DNBs) were generated with the ssDNA circles by rolling the circles during replication to significantly increase the fluorescent signals during the sequencing process. The DNBs were loaded into the patterned nanoarrays and sequenced on the Nova-seq6000 sequencing platform with a paired end read length of 28–150 bp.

### Single-cell RNA-seq data processing

We firstly processed 10× Genomics raw data using the Cell Ranger Single-Cell Software Suite (version 4.0.0), including using ‘cellranger mkfastq’ to demultiplex raw base call files into FASTQ files, then using ‘cellranger count’ to perform alignment, filtering, barcode counting, and UMI counting. The reads were aligned to the axolotl reference genome AmbMex60DD (Schloissnig et al., 2021) (https://www.axolotl-omics.org/assemblies) with STAR (Dobin et al., 2013) which was imbedded in the 10× Genomics Cell Ranger-4.0.0 software with default parameters to generate the absolute Unique Molecular Identifier (UMI) counts. The output from different lanes was finally aggregated using “cellranger aggr” with the default parameter setting. Then we mapped UMIs to genes, followed by removing low-quality cells. Cells with fewer 200 unique genes were excluded for further analysis. Then we utilized functions in the Seurat package to normalize and scale the single-cell gene expression data. The data were first normalized using the “NormalizeData” function with the normalization method set to “LogNormalize.” Specifically, the expression of each gene *i* in cell *j* was determined by the UMI count of gene *i* divided by the total number of UMI of cell *j*, followed by multiplying by 10,000; for the normalization the log-transformed counts were then computed with a base of 2. We then removed the irrelevant sources of variation by regressing out cell-cell variation within gene expression driven by batch and the number of detected UMI; this was implemented using the “ScaleData” function. Finally, the corrected expression matrix was used as an input for further analysis.

### Dimension reduction, cell clustering

To enhance the identification of common cell types and enable comparative analyses of different experiments, we integrated datasets from all stages by using the “FindIntegrationAnchors” function in Seurat, which was implemented in the Seurat workflow (https://satijalab.org/seurat/v3.0/immune_alignment.html).

We next restricted the corrected expression matrix to the subsets of highly variable genes (HVGs), and then centered and scaled values before performing dimension reduction and clustering on them. Methodologically, the top 2,000 HVGs in single-cell data were selected by first fitting a generalized linear model to the mean-dependent trend for the gene-specific variance of all genes, and selecting genes that deviated significantly from the fitted curve. This was implemented using the “FindVariableGenes” function in the Seurat package by setting the valid value of average expression as a range from 0.05 to 5 and that of dispersion as no less than 0.5.

We then used the “RunPCA” function in the Seurat package to perform principal component analysis (PCA) on the single-cell expression matrix with genes restricted to HVGs. Given that many principal components explain very low proportion of the variance, the signal-to-noise ratio can be improved substantially by selecting a subset of significant principal components. The number of significant principal components was determined by the permutation test, implemented using the permutationPA function from the jackstraw R package (https://cran.r-project.org/web/packages/jackstraw). We then utilized the “FindClusters” function in the Seurat package to conduct the cell clustering analysis through embedding cells into a graph structure in PCA space. Due to the large number of cells in our study, we set the parameter resolution to 0.8. This identified a total of 41 clusters. The clustering results were presented using a uniform manifold approximation and projection (UMAP) based on plots generated by the “RunUMAP” function. The “FindAllMarkers” function was used to identify differentially expressed genes with default parameters. Finally, top 100 differentially expressed genes in each cluster were kept as marker genes of each cluster.

### Correlation and differentially expressed genes analysis

To calculate the correlation of each cell type between controls and *Pax7* mutants, we firstly calculated the average expression of each gene to construct a pseudobulk gene expression matrix. Then the Pearson correlation coefficient of each cell type between controls and *Pax7* mutants was calculated and visualized by R package “pheatmap.”

To obtain the differentially expressed genes between each cell type between controls and *Pax7* mutants, the “FindMarkers” function in the Seurat package was used. The differentially expressed genes need to meet the threshold of adjust *p*-value < 0.05 and |log2 (Fold Change)| > 1.

### Identification of cell types

To obtain the comprehensive genetic annotation information, we reannotated the gene annotation of axolotl using the protein sequence. The human (GRCh38) and mouse (GRCm38) protein sequences were downloaded from the NCBI. We then aligned all the public data against the axolotl protein sequence by using all vs. all Blast (Altschul et al., 1990) (version 2.2.25) with the main parameter of “–e 1e-5.” The best hit result from alignments with a Z-score ≥ 200 was used to perform the homologous annotation. Then, top 100 marker genes in each cluster were annotated manually by querying literatures one by one to define the cell types.

### Quantifications and statistical analyses

All quantifications were carried out manually. For each sample, we collected a series of 10 μm limb sections for immunohistochemistry and quantification. The adjacent sections have an 80 µm interval. For control and *Pax7* mutant animals at 0, 4, 9, 16 dpa, limb samples were longitudinal sectioned. Generally, each control and *Pax7* mutant sample contains 11–14 and 7–9 sections, respectively. All relevant cells in sections of newly regenerated blastema and within 500 μm zone proximal to the amputation plane were quantified. In addition, cross sections were collected from 47-days fully regenerated control and *Pax7* mutant limb samples. Cells in the region of interest from this series of sections were quantified and summed for statistics. All data are presented as mean ± sem, an unpaired *t*-test was used to determine whether differences in the experimental group were statistically significant. Student’s *t*-tests were performed using Graphpad Prism 9.0.0. A *p*-value less than 0.05 was considered to be statistically significant.

## Data Availability

The data presented in the study are deposited in the NCBI repository, accession number PRJNA877838.
