# Supplementary figures and images for: Muscles are barely required for the patterning and cell dynamics in axolotl limb regeneration
Source: Front Genet. 2022 Oct 10;13:1036641. doi: 10.3389/fgene.2022.1036641 (PMC9589296; doi:10.3389/fgene.2022.1036641)

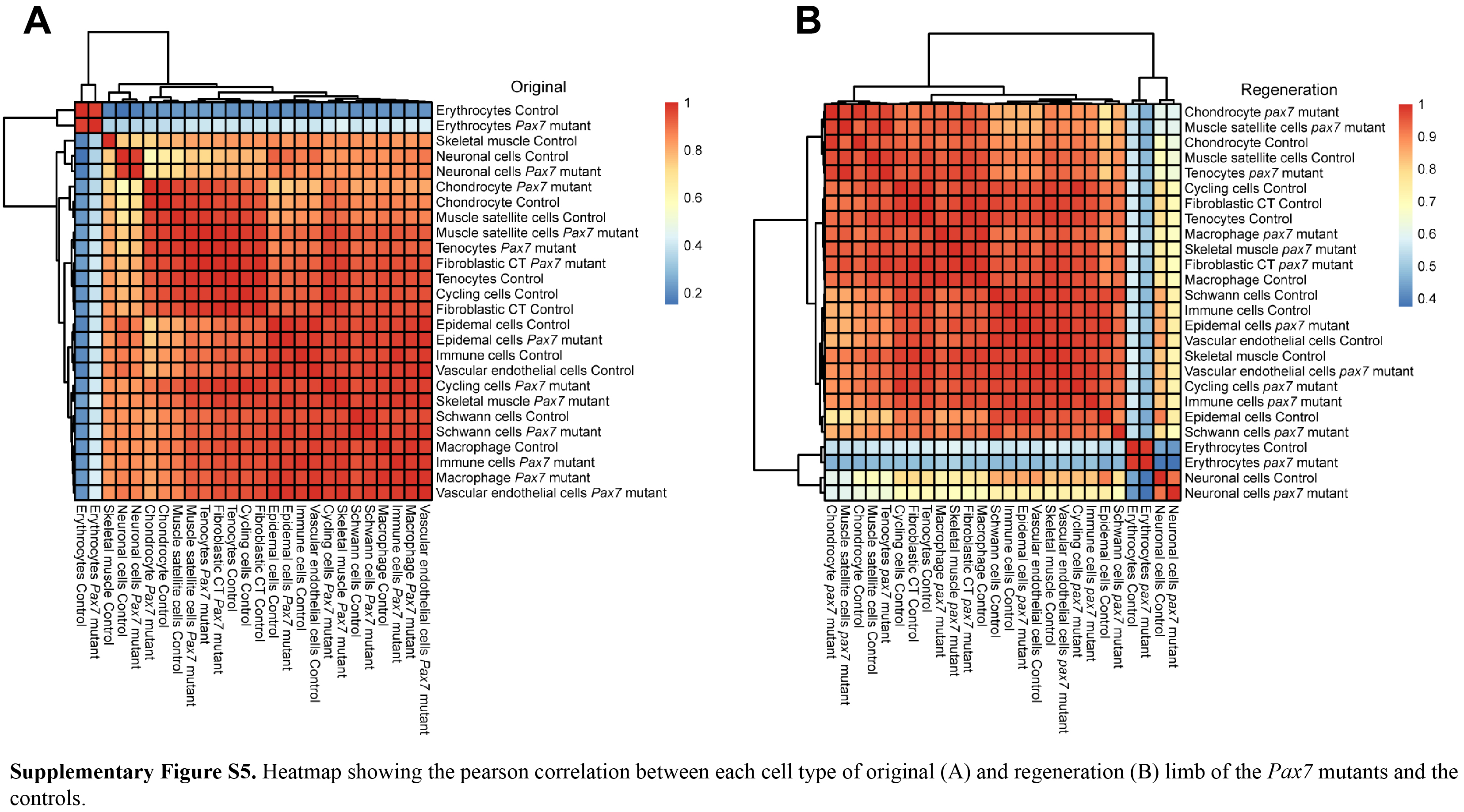

Supplement: Supplementary file 1 [file Image5.jpg]

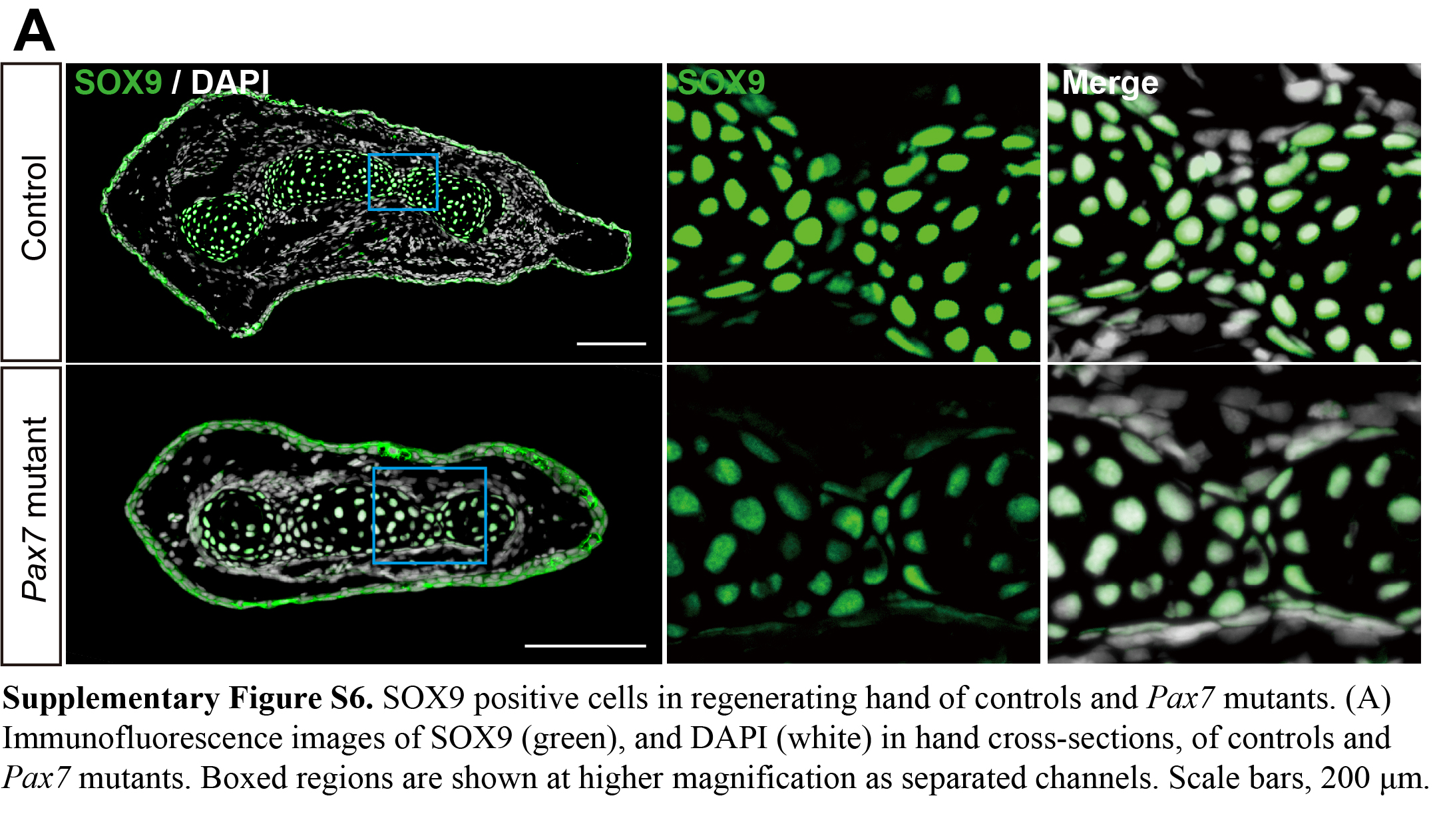

Supplement: Supplementary file 2 [file Image6.jpg]

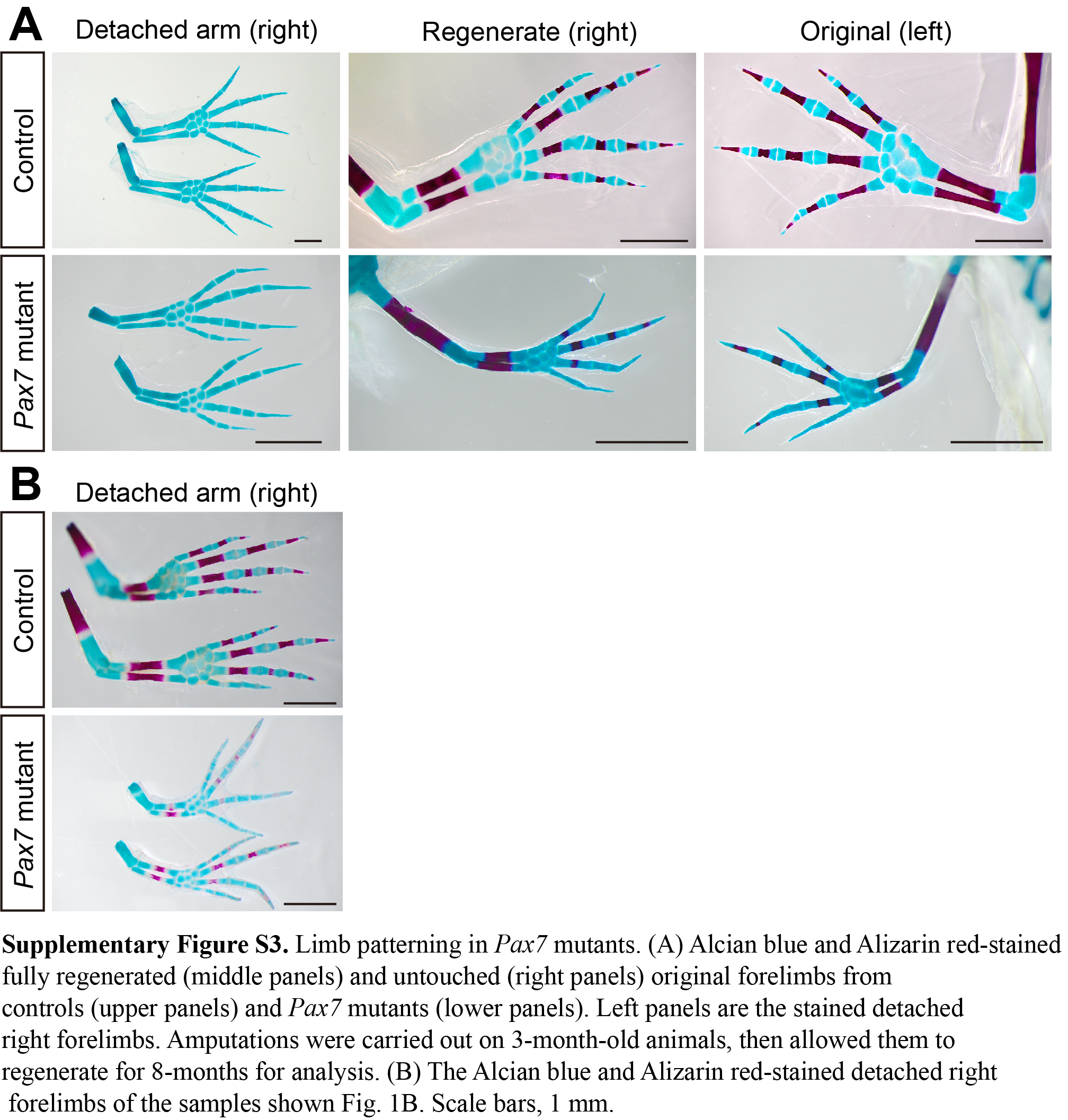

Supplement: Supplementary file 3 [file Image3.jpg]

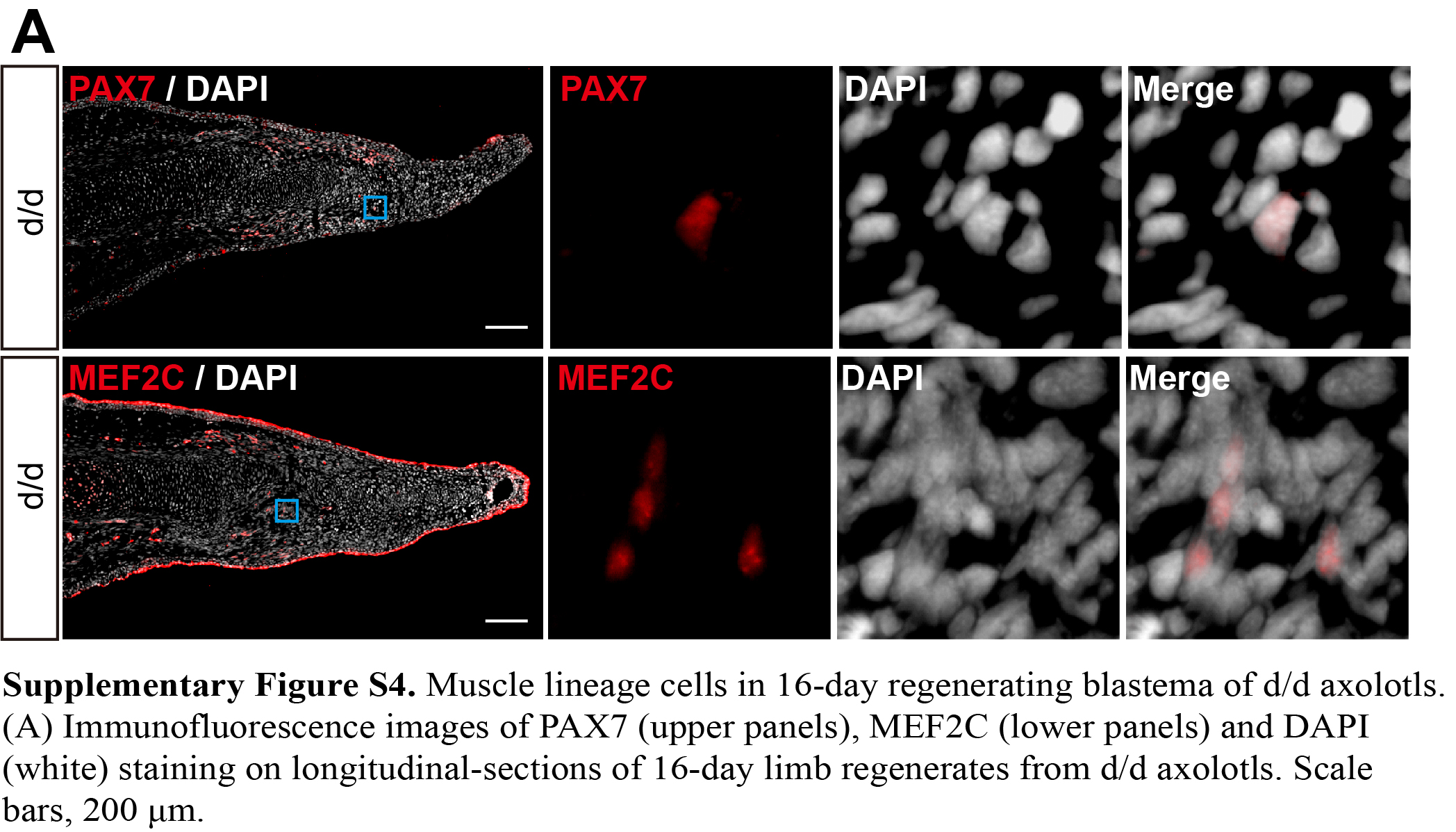

Supplement: Supplementary file 4 [file Image4.JPEG]

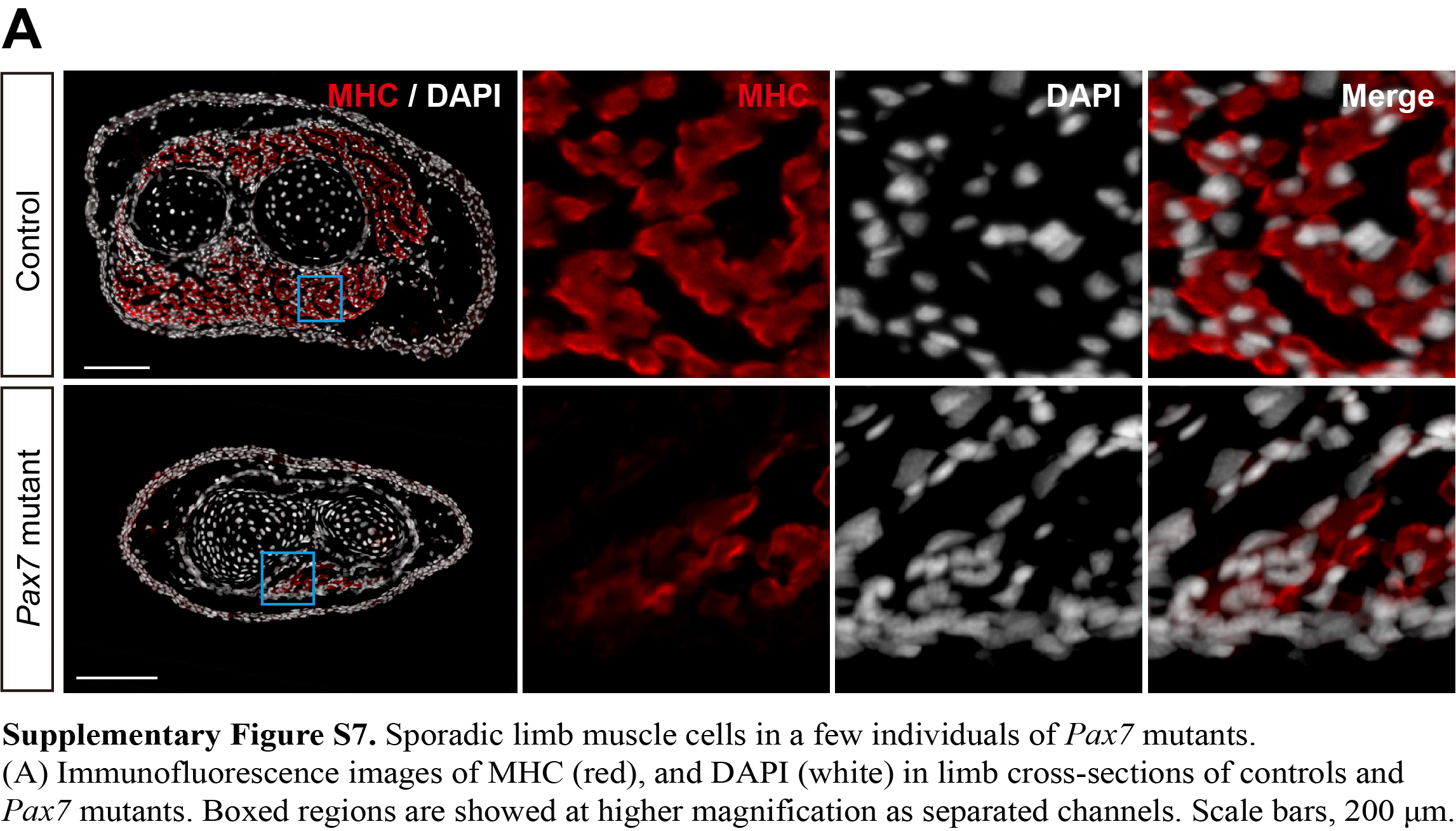

Supplement: Supplementary file 5 [file Image7.JPEG]

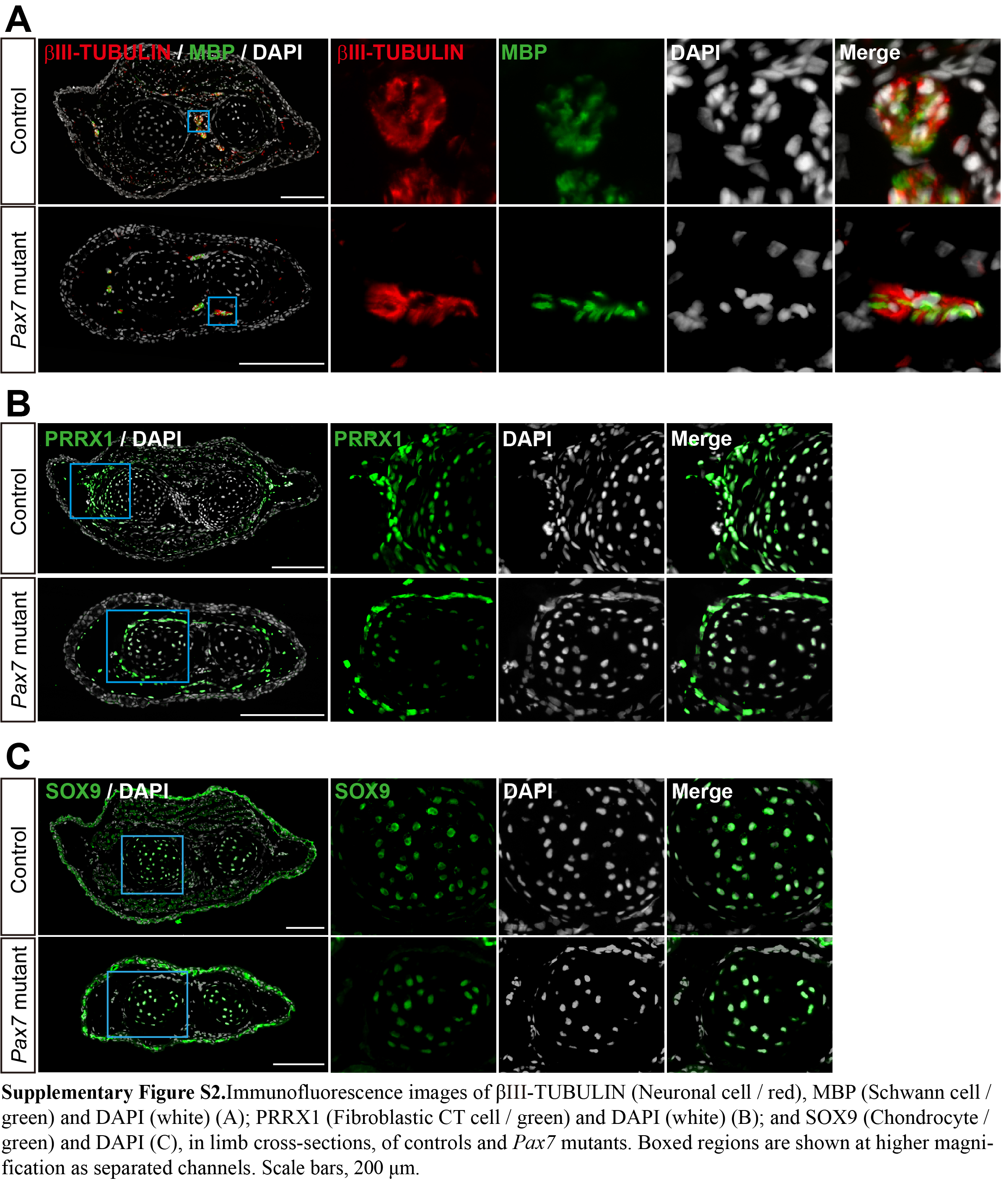

Supplement: Supplementary file 6 [file Image2.JPEG]

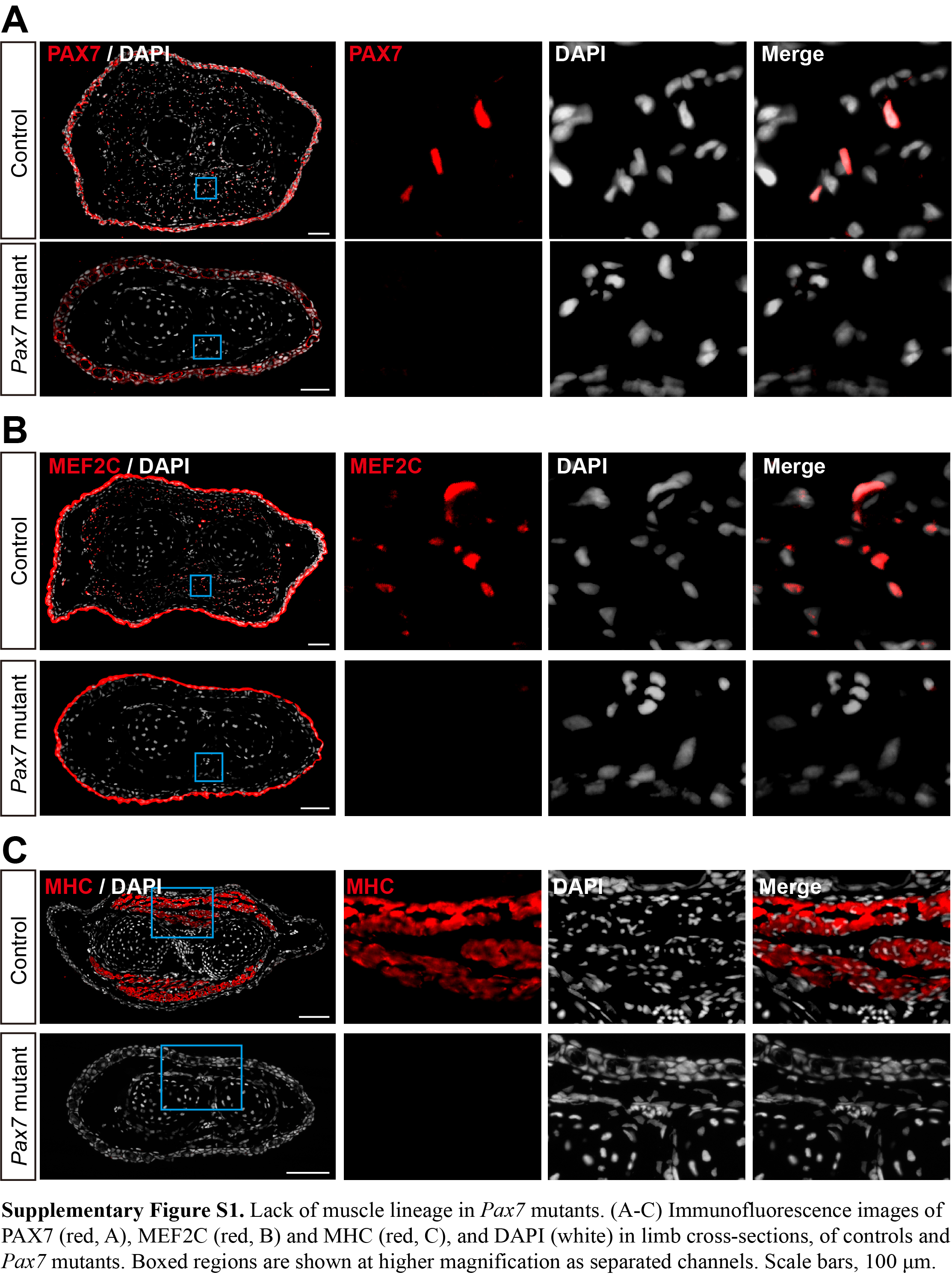

Supplement: Supplementary file 7 [file Image1.jpg]
