# Supplementary material for: Muscles are barely required for the patterning and cell dynamics in axolotl limb regeneration
Source: Front Genet. 2022 Oct 10;13:1036641. doi: 10.3389/fgene.2022.1036641 (PMC9589296; doi:10.3389/fgene.2022.1036641)
Supplement: Supplementary file 8 [file DataSheet1.docx]

**Supplementary Table S1. The number of fingers at 42 days post amputation.**

| Groups | Number of three fingers animals | Number of four fingers animals | Number of animals counted |
| --- | --- | --- | --- |
| Control | 0 (0%) | 7 (100%) | 7 |
| *Pax7* mutant | 4 (57%) | 3 (43%) | 7 |

**Supplementary Table S2. The number of limbs used for single cell dissociation and cells loaded for sc-RNA sequencing in each group.**

| Groups | Number of limbs used for single cell dissociation in each group | Number of cells loaded for sc-RNA sequencing in each group |
| --- | --- | --- |
| Control original | 30 | 30,282 |
| Control regeneration | 30 | 30,326 |
| *Pax7* mutant original | 55 | 30,350 |
| *Pax7* mutant regeneration | 55 | 30,576 |
